# Motivation toward Physical Exercise and Subjective Wellbeing: The Mediating Role of Trait Self-Control

**DOI:** 10.3389/fpsyg.2016.01546

**Published:** 2016-10-05

**Authors:** Walid Briki

**Affiliations:** Sport Science Program, College of Arts and Sciences, Qatar UniversityDoha, Qatar

**Keywords:** physical activity, self-determined motivation, self-regulation, self-control, psychological health

## Abstract

Motivation toward physical exercise (MPE) and trait self-control (TSC) were identified as key predictors of subjective wellbeing (SWB). However, there has not been any research designed to examine the mediating role of TSC in the relationship between MPE and SWB. The present study utilizes self-determination theory, control-process theory of self-regulation, and theory of multiple pathways of TSC in order to examine whether TSC mediates the relationships of autonomous MPE (A-MPE), controlled MPE (C-MPE), and impersonal MPE (NO-MPE) with SWB using structural equation modeling (XLSTAT PLS). Three hundred seventeen adult American individuals (*M*_age_ = 32.97, *SD*_age_ = 11.30), who reported to be regular exercisers, voluntarily answered questionnaires assessing MPE, TSC, and SWB. Correlational analyses revealed positive relationships between A-MPE, TSC, and SWB, and negative relationships of C-MPE and NO-MPE with TSC and SWB. Mediation analyses revealed that TSC mediated the relationships of A-MPE (partial mediation) and C-MPE (full mediation) with SWB, but did not mediate the relationship between NO-MPE and SWB. The estimates of the quality of the hypothesized model were acceptable (outer model GoF = 0.935; absolute GoF = 0.330; relative GoF = 0.942; inner model GoF = 1.008; *R*^2^ = 36.947%). Finally, this study supports the view that MPE can influence SWB through TSC, and incites to pursue the examination of the relationships between self-determined motivation, self-regulation mechanisms, and health-related outcomes.

## Introduction

Subjective wellbeing (SWB), which can be defined as “…people’s evaluations of their lives – the degree to which their thoughtful appraisals and affective reactions indicate that their lives are desirable and proceeding well” (Diener et al., 2015, p. 234), represents a growing subject of interest around the world for psychologists, economists, philosophers, and politicians (e.g., Diener, 2000; Diener et al., 2015). Such a phenomenon would reflect the tendency of most societies to recognize the value of the human being and the importance of taking into account self-perceptions for evaluating individual’s life (e.g., Diener et al., 2015). Furthermore, the study of SWB lies in positive psychology that corresponds to the scientific study of positive psychological outcomes, psychological health, human capacities, and processes leading to fostering optimal functioning and development (e.g., Peterson and Park, 2003; Peterson and Seligman, 2004). In that perspective, research has found that SWB was a key predictor of health, longevity, moral behavior, and performance (e.g., De Neve et al., 2013). For that reason, examining the predictive factors of SWB is of great importance. In addition, such a subject may represent a considerable interest to a worldwide audience.

Research has found that performing physical exercise regularly could promote health and SWB (e.g., Scully et al., 1998; Melzer et al., 2004; Puetz et al., 2006; Sjögren et al., 2006; Meyers, 2008; Biddle and Asare, 2012). Physical exercise represents a leisure-time physical activity, and can be defined as “…cumulative, acute bouts of physical activity that are planned, structured, and repeated and result in improvement or maintenance of one or more components of physical fitness, including cardiorespiratory capacity, muscle strength, body composition, and flexibility” (Puetz et al., 2006). Exercise psychologists have recently examined the relationship between the different types of commitment to exercise – operationalized through the notion of motivation toward physical exercise (MPE) – and SWB (e.g., Sebire et al., 2009; Gillison et al., 2011). Globally, they evidenced that the type of MPE (autonomous vs. controlled) could influence differently SWB. However, the mechanisms underlying such effects are still unclear and require additional investigations (e.g., Standage and Ryan, 2012). Self-control, which can be conceived as the effortful control and effortless (automatic) forms of goal-directed behavior (e.g., Hagger, 2013, 2014), would represent one of the most adaptive variables of the human psyche (e.g., Carver and Scheier, 1998; Tangney et al., 2004) and was found to play a key role in the development of SWB (e.g., Hofmann et al., 2014). Additionally, the type of motivation for goal pursuit appeared to affect the effectiveness of self-control processes (e.g., Milyavskaya et al., 2015). Therefore, the present article aims at examining *whether* the type of MPE might influence SWB through self-control.

### Self-Determined Motivation and Subjective Wellbeing

Research focused on MPE frequently used the conceptual framework of self-determination theory (e.g., Deci and Ryan, 1985, 2000, 2008a,b). This theory is grounded in an organismic approach that considers that individuals are, by nature, active, curious, self-motivated, vital, and enthusiastic, even if they may display passivity, laziness, anxiety, or depression symptoms. Interestingly, the theory posits that individuals’ positive or negative functioning would result from their interaction with the social environment that either supports or thwarts their deep nature, respectively. Specifically, the theory posits that the human psyche is characterized by three innate needs – i.e., needs for competence (i.e., need of being able to perform well something), relatedness (i.e., need of being connected with others), and autonomy (i.e., need of being at the origin of one’s own behavior) – that serve the function of developing optimal functioning, performance, and wellbeing. The theory distinguishes three types of functioning: Autonomous, controlled, and impersonal regulations (or motivations).

Autonomous motivation reflects “…a motivational state in which self-initiation and coordination of personally endorsed behaviors predominate” (Weinstein et al., 2011, p. 527). A social environment capable of satisfying the three innate needs is presumed to develop a strong sense of autonomous motivation, which is supposed to be related to optimal functioning, performance, and wellbeing. Intrinsic regulation (i.e., action is based on personal interest and satisfaction), integrated regulation (i.e., action is consistent with different aspects of the self), and identified regulation (i.e., action is personally valued and important) represent forms of autonomous motivation (e.g., Standage and Ryan, 2012). Controlled motivation reflects a “…functioning driven by externally imposed and introjected contingencies, eliciting pressure to conform to perceived expectations” (Weinstein et al., 2011, p. 527). It is assumed that a social environment that is capable of satisfying the needs for competence and relatedness but that thwarts the need for autonomy is presumed to develop a strong sense of controlled motivation. This type of motivation is supposed to be associated with a rigid functioning and a decreased wellbeing. Introjected regulation (i.e., action responds to the desire to avoid guilt and shame or to develop feelings of worth) and external regulation (i.e., action responds to the desire to obtain reward, to avoid punishment, or to meet external expectations) refer to controlled forms of motivation (e.g., Standage and Ryan, 2012). Impersonal motivation (or amotivation), which is associated with poor functioning, passivity and depressed symptoms, develops when the social environment thwarts the three needs (e.g., Standage and Ryan, 2012).

Grounded in the self-determination theory, empirical investigations have reported associations between the different types of motivation and constructs related to SWB (e.g., Gillison et al., 2006; Sebire et al., 2009). Specifically, it was found that autonomous forms of MPE (A-MPE) were positively related to: (a) SWB (measured through a composite construct combining subjective vitality and happiness, *r* = 0.29, Sebire et al., 2009), (b) quality of life (measured through a composite construct combining 10 health-related variables, β = 0.37, Gillison et al., 2006), (c) body satisfaction (*r*s = 0.28–0.34, Gillison et al., 2011), and (d) physical self-worth (*r*s = 0.24–0.36, Thøgersen-Ntoumani and Ntoumanis, 2006; Sebire et al., 2009). Authors also revealed that A-MPE was negatively related to exercise anxiety (*r* = -0.33, Sebire et al., 2009) and social physique anxiety (*r*s = -0.22 to -0.11, Thøgersen-Ntoumani and Ntoumanis, 2006; Gillison et al., 2011). In contrast, controlled forms of MPE (C-MPE) and impersonal MPE (NO-MPE) were associated with maladaptive psychological outcomes (e.g., Thøgersen-Ntoumani and Ntoumanis, 2006). Specifically, forms of C-MPE appeared to be positively related or unrelated to social physique anxiety (*r*s = 0.10–0.26, Thøgersen-Ntoumani and Ntoumanis, 2006; Gillison et al., 2011). On the other hand, they appeared to be negatively related to body satisfaction (*r* = -0.16, Gillison et al., 2011) and SWB (measured through subjective vitality, β = -0.81, Edmunds et al., 2006), and negatively related or unrelated to physical self-worth (*r*s = -0.19 to 0.01, Thøgersen-Ntoumani and Ntoumanis, 2006). NO-MPE was positively related to social physique anxiety (*r*s = 0.18–0.21, Thøgersen-Ntoumani and Ntoumanis, 2006; Gillison et al., 2011) and negatively related to body satisfaction (*r* = -0.19, Gillison et al., 2011) and physical self-worth (*r* = -0.18, Thøgersen-Ntoumani and Ntoumanis, 2006).

Finally, there is strong evidence that A-MPE (or NO-MPE) can promote (or hinder) positive feelings. However, the detrimental effect of C-MPE on positive feelings lacks of consistent evidence in the literature. In the present study, we present some reasons to argue that the relationship between self-determined motivation and positive feelings might be explained, at least partly, by the intervention of self-regulation mechanisms, such as trait self-control (TSC).

### Self-Determined Motivation and Trait Self-Control

Self-regulation reflects self-corrective adjustments that occur to be on track to attain the desired goal (e.g., Carver and Scheier, 1998), and self-control represents a crucial component of this broader phenomenon. Generally, self-control reflects the ability to operate changes in the self so as to develop an optimal adjustment between the self and the world (Tangney et al., 2004). More specifically, self-control can be defined as the capacity of the self to override prepotent responses and to regulate affects, cognitions, and behaviors (e.g., Baumeister and Heatherton, 1996; Tangney et al., 2004; Baumeister et al., 2007). For example, people with high TSC would be better at suppressing one goal (e.g., eating a palatable food) than people with low TSC to pursue another one (e.g., controlling one’s weight) that is viewed to have greater importance or utility (e.g., Stroebe et al., 2013). Additionally, authors agree that self-control corresponds to a reservoir of limited resources designed to promote helpful responses (e.g., making plans) and inhibit unhelpful responses (e.g., inhibiting temptations) (e.g., Baumeister and Heatherton, 1996; Muraven, 2008; Hagger, 2013). For example, people with high TSC would have more available resources to self-regulate than people with low TSC.

According to the control-process theory of self-regulation (Carver and Scheier, 1990, 1998), the effectiveness of self-regulation mainly depends upon one’s capacity to pursue clear goals. A strong adherence to a specific activity leads to operate a goal selection depending on the degree of *relevance* and *importance* of goals for the self. For example, in the case of physical exercise, such a goal selection could consist, on the one hand, in adopting certain goals – such as going to bed early, eating a balanced diet, etc., – and, on the other hand, in eschewing other goals – such as drinking alcohol, smoking, etc. Deci and Ryan (2000) considered that goal-directed activities might differ in the degree to which they are autonomous (i.e., enacted with a full sense of volition and choice) or controlled (i.e., enacted with a full sense of being pressured and controlled). Autonomous activities are consistent with one’s integrated sense of self, whereas controlled activities have not been assimilated to the self and, thus, remain external to the self. Accordingly, people who pursue autonomous activities, relative to those who pursue controlled activities, would be more likely to operate a goal selection in accordance with their activity. In that regard, autonomous motivation would be associated with better self-regulation processes than controlled motivation.

Research has revealed that exerting self-control for autonomous reasons was less depleting than exerting self-control for controlled reasons, allowing people to perform better on a subsequent task (Muraven et al., 2007, 2008; Muraven, 2008). Similarly, Moller et al. (2006) found that making choices for autonomous reasons were less depleting and led to better self-control performance than making choices for controlled reasons. This pattern of results suggest that autonomous motivation, relative to controlled motivation, would save more resources for subsequent tasks, thereby leading to maintaining focus on relevant goals. An important implication of such results is that autonomous self-control refers to the identification and integration of values, goals, and behavioral regulations, thereby leading to reinforcing self-control (Deci and Ryan, 1987).

Focusing on mechanisms of self-control, studies have found that autonomous motivation was globally associated with enhanced self-control. More specifically, autonomous motivation appeared to be positively related to TSC (*r* = 0.22, Briki et al., 2015) and implementation planning (*r*s = 0.15–0.31, Koestner et al., 2008), and positively related or unrelated to automatic attraction toward helpful goals (e.g., healthy foods; βs = -0.05 to 0.33, Milyavskaya et al., 2015). However, it appeared to be negatively related to automatic attraction toward temptations (e.g., highly palatable, but unhealthy foods; βs = -0.25 to -0.17, Milyavskaya et al., 2015) and perception of encountering obstacles (βs = -0.25 to -0.22, Milyavskaya et al., 2015). Autonomous motivation appeared to be unrelated to controlled attraction toward helpful and unhelpful goals (βs = 0.01–0.09, Milyavskaya et al., 2015) and perception of effort (β = 0.03, Milyavskaya et al., 2015). Using functional magnetic resonance imaging, Lopez et al. (2016) observed positive associations between autonomous motivation, positive mood, and the activity of inferior frontal gyrus – a brain zone reputed to be associated with inhibitory control –, suggesting that autonomous motivation would particularly involve the inhibition of goal-disruptive temptations. Furthermore, controlled motivation was globally unrelated to self-control (e.g., Briki et al., 2015; Milyavskaya et al., 2015). More specifically, it appeared to be unrelated to TSC (*r*s = 0.09–0.14, Briki et al., 2015), implementation planning (*r*s = 0.01–0.05, Koestner et al., 2008), automatic attraction toward helpful and unhelpful goals (βs = -0.05 to 0.19, Milyavskaya et al., 2015), and controlled attraction toward helpful goals (β = -0.05, Milyavskaya et al., 2015). However, controlled motivation was found to be positively related to perception of encountering obstacles (β = 0.28, Milyavskaya et al., 2015).

Finally, the set of these studies suggest that autonomous motivation would entail a sense of self-control (e.g., TSC) by inhibiting automatically goal-disruptive impulses and implementing helpful strategies. However, the results reveal unclear relationships between controlled motivation and mechanisms of self-control.

### Trait Self-Control and Subjective Wellbeing

Does TSC make people happy? A set of studies have provided evidence about the beneficial effects of TSC on positive affect and SWB. The results of De Ridder et al.’s (2012) meta-analysis revealed a positive relationship between TSC and SWB [measured through different types of construct, such as self-esteem, happiness, or absence of depression; *r* = 0.33 (number of used studies = 16)]. In the same vein, authors reported that TSC was positively related to SWB (measured through satisfaction with one’s life or happiness, *r*s = 0.24–0.50, Cheung et al., 2014; Hofmann et al., 2014; Briki et al., 2015), general self-worth (*r* = 0.25, Briki et al., 2015), and positive affect (*r*s = 0.27–0.35, Hofmann et al., 2014; Briki et al., 2015). With a particular focus on the exercise setting, authors showed a positive relationship (*r* = 0.20) between conscientiousness (reflecting the notion of self-control) and positive affective attitude (measured through different constructs, such as enjoyment, interest, and calmness; Rhodes et al., 2002).

Why does TSC make people happy? To answer that question, it is necessary to understand how TSC can predict goal-directed behavior. Drawing from empirical and theoretical work, Hagger (2013, 2014) proposed a theory delineating three types of pathway by which TSC influences goal-directed behavior (i.e., direct, indirect, and interactive effects). The first pathway (called “P1”) corresponds to a direct relationship between TSC and behavior. For example, people with high TSC are more likely to display goal-directed behavior than people with low TSC. The second and third pathways (called “P2” and “P3”) correspond to indirect pathways mediated by motivational components. Specifically, P2 reflects an effect on goal-directed behavior mediated by intention, in the sense that people are more likely to develop intentions to reach a goal that promote, in turn, subsequent goal-directed behavior. P3 corresponds to an effect on goal-directed behavior mediated by impulsive motives, indicating that people are more likely to borrow impulsive route to action in which behavior is controlled by more spontaneous processes. The fourth and fifth pathways (called “P4” and “P5”) correspond to two interactive pathways in which TSC (and, especially, available resources) moderates the relationship between motivational components and behavior. Specifically, P4 and P5 represent the processes by which self-control resources influence the conversion of intentions and impulsive motives into action, respectively. In sum, people with high TSC would be not only more likely to develop plans for action in order to attain a self-relevant goal (P2), but also more likely to convert such reasoned decisions into action (P4). In addition, they would be less likely to fall under the control of impulses (P3) and more likely to suppress such impulses (P5).

In line with that theory that predicts that TSC stimulates plans for action and their conversion into action (Hagger, 2013, 2014), authors have demonstrated that TSC could foster happiness and SWB by stimulating effective strategies (Cheung et al., 2014). More specifically, Cheung et al. (2014) revealed a positive relationship between TSC and promotion focus (i.e., motivational orientation concerned with growth, advancement, and accomplishment; *r* = 0.21), and a negative relationship between TSC and prevention focus (i.e., motivational orientation concerned with vigilance, responsibility, and ought; *r* = -0.48). Additionally, they found that both motivational orientations mediated partially the relationship between TSC and happiness, indicating that TSC positively predicted happiness directly and indirectly through increased promotion focus and decreased prevention focus. Furthermore, and consistent with the view that TSC can inhibit goal-disruptive impulses (Hagger, 2013, 2014), authors have revealed that the beneficial effects of TSC on positive affect and SWB might be due to the capability of TSC to manage conflict between competing goals and desires (Hofmann et al., 2012, 2014). More specifically, Hofmann et al. (2014) revealed that TSC was negatively related to problematic desires (*r*s = -0.18 to -0.20; Hofmann et al., 2012, 2014) and that people with high TSC experienced less frequently problematic desires than people with low TSC. Interestingly, Hofmann et al. (2014) also revealed that positive affect (reflecting a momentary psychological state) positively predicted SWB (reflecting satisfaction with life in general), and the authors interpreted this result as the reflection of the tendency of TSC to reinforce consistency within the self, leading to promote positive experiences.

In sum, the set of results suggest that TSC would promote happiness and SWB by activating helpful plans for action and inhibiting problematic desires, leading to increase satisfaction and the likelihood of goal attainment. In that regard, happiness and SWB would be the product of the combination of increased consistency within the self and successful experiences.

### The Mediating Role of Trait Self-Control

McCullough and Willoughby (2009) suppose that TSC represents not only the key variable of self-regulation processes, but also one of the most important mediating variables to account for the relationship between motivation and psychosocial outcomes. Several studies conducted in different domains, such as health, education, and leisure, have evidenced such a hypothesis (e.g., Koestner et al., 2008; Briki et al., 2015; Milyavskaya et al., 2015). More specifically, authors examined the mediating role of self-control components (e.g., implementation planning, automatic attraction toward temptations) in the relationship between self-determined motivation and perceptions of obstacles or goal progress (Koestner et al., 2008; Milyavskaya et al., 2015). They observed that autonomous motivation positively predicted goal progress through increased implementation planning, and that the implementation of approach-oriented plans (i.e., intentions to move toward desired outcomes) strengthened that relationship (moderating effect; Koestner et al., 2008). They also observed that autonomous motivation negatively predicted perception of obstacles through decreased attraction toward goal-disruptive temptations (Milyavskaya et al., 2015), and that controlled motivation negatively predicted goal process through increased perception of obstacles (Milyavskaya et al., 2015). Focusing on wellbeing, Briki et al. (2015) have recently examined the relationships between identified religiosity (reflecting autonomous motivation), introjected religiosity (reflecting controlled motivation), TSC, and SWB. The authors showed that autonomous motivation positively predicted SWB directly and indirectly through enhanced TSC. By contrast, TSC did not mediate the relationship between controlled motivation and SWB, supporting previous results showing an independence relationship between controlled motivation and self-control (Koestner et al., 2008; Milyavskaya et al., 2015).

### Research Overview

**FIGURE 1:**
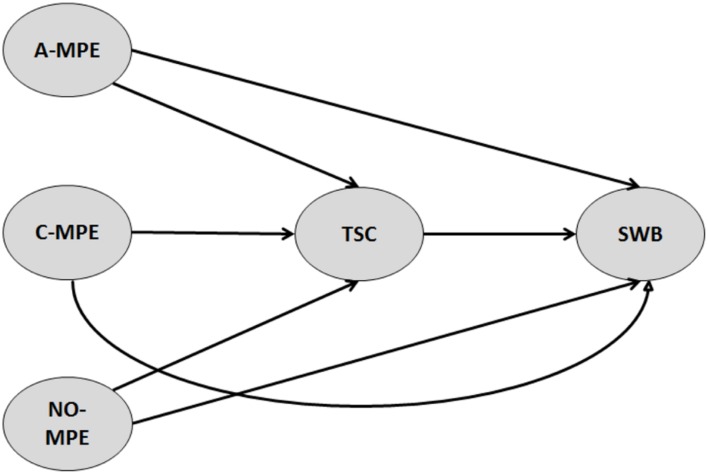
**Conceptual diagram of the model**. A-MPE, autonomous motivation toward physical exercise; C-MPE, controlled motivation toward physical exercise; NO-MPE, impersonal motivation toward physical exercise; TSC, trait self-control; SWB, subjective wellbeing.

The present study attempted to examine how MPE, TSC, and SWB might be interrelated, and whether TSC might mediate the relationships of A-MPE and C-MPE with SWB (assessed through the happiness and vitality subscales) in adult regular exercisers. To do so, we built and examined a model using the structural equation model analysis (**Figure 1**). To assess its quality, different sorts of index were used, such as the goodness-of-fit (GoF) index (e.g., absolute GoF, relative GoF) and the coefficient of determination of the endogenous latent variables (*R*^2^) (e.g., Henseler et al., 2009; Vinzi et al., 2010). The higher the values of GoF indexes and R^2^, the better the model (see the “Analysis” section below). Based on the tenets of the self-determination theory (e.g., Deci and Ryan, 2000, 2008a,b), the control-process theory of self-regulation (e.g., Carver and Scheier, 1990, 1998; McCullough and Willoughby, 2009), and the theory of multiple pathways of TSC (Hagger, 2013, 2014), and consistent with the above-mentioned studies’ results, the present study proposes four sets of hypothesis:

#### Relationships between MPE and SWB

In line with previous studies showing that A-MPE (or NO-MPE) was positively (or negatively) related to positive psychological outcomes (e.g., SWB) (e.g., Gillison et al., 2006, 2011; Thøgersen-Ntoumani and Ntoumanis, 2006; Sebire et al., 2009), we expect A-MPE (or NO-MPE) to be positively (or negatively) related to SWB. However, because research has found that C-MPE was negatively related or unrelated to positive psychological outcomes (e.g., Thøgersen-Ntoumani and Ntoumanis, 2006), we do not have any prediction concerning the relationship between C-MPE and SWB.

#### Relationships between MPE and TSC

Consistent with previous studies showing positive relationships between autonomous motivation and self-control (e.g., Koestner et al., 2008; Briki et al., 2015), we expect A-MPE to be positively related to TSC. However, no clear or consistent evidence allow us to formulate any expectations regarding the relationships between C-MPE and TSC and between NO-MPE and TSC.

#### Relationship between TSC and SWB

Because research has found that self-control fostered positive affect and SWB (e.g., Cheung et al., 2014; Hofmann et al., 2014), we expect TSC to be positively related to SWB.

#### Mediations

Consistent with previous studies indicating that self-control-related-variables (e.g., TSC, implementation planning) might mediate the relationship between autonomous motivation and positive perceptions or feelings (Koestner et al., 2008; Briki et al., 2015), we expect TSC to mediate the relationship between A-MPE and SWB. No consistent evidence allow us to formulate any predictions about the mediating role of TSC in the relationships between C-MPE and SWB and between NO-MPE and SWB.

## Materials and Methods

### Participants

Three hundred seventeen adult American volunteers (223 females, 70.3%, and 94 males, 29.7%; *M*_age_ = 32.97, *SD*_age_ = 11.30; *M*_size_ = 1.64 m, *SD*_size_ = 0.18; *M*_weight_ = 81.24 kg, *SD*_weight_ = 29.14), who reported to perform physical exercise regularly (e.g., walking, swimming, biking), were recruited from a popular crowdsourcing on-line platform (ClickWorker^1^). On average, the participants reported to perform physical exercise 3.84 times a week (*SD* = 1.53) since 5.70 years (*SD* = 7.73). This sample included participants who were heterogeneous on several qualitative variables, such as socio-demographic variables, (i.e., ethnicity, professional status, familial status), medical variables (i.e., chronic mental and physical disease), and exercise-related variables (i.e., intensity, duration, and mode; **Table 1**).

**Table 1:** Socio-demography and medical situation of participants, as well as characteristics of their physical exercise.

	*n*	%
Socio-demography:		
Ethnicity:		
African American	58	18.3
Asian American	22	6.9
Caucasian American	203	64
Hispanic American	22	6.9
Other	12	3.8
Familial status:		
Living in family	274	86.4
Professional status:		
Working	104	32.8
Medical situation:		
Physical disease:		
Having a chronic disease	40	12.6
Psychological disease:		
Having a chronic disease	42	13.2
Living in wheelchairs:		
No	317	100
Exercise characteristics:		
Duration:		
0–30°min	101	31.9
31–60°min	167	52.7
61–90°min	39	12.3
91–120°min	10	3.2
Mode:		
Alone	199	62.8
With friends/partner/family	81	25.6
Within a guided program	37	11.7
Intensity:		
Aerobic exercise	236	74.4
Anaerobic exercise	81	25.6

### Study Design and Procedure

The present study design was submitted and approved by the ethics committee of Qatar University. Each participant provided his/her informed written consent. The setup of this study included a form that was accessible to participants via a specific web address. This form included general information about the study, a consent form, and questionnaires (see the “Measures” section below). Before answering questions, participants were told that: (a) the survey was designed to examine relationships between physical exercise and feelings, (b) they had to perform physical exercise regularly to participate in that survey, and (c) their responses to the survey would be completely anonymous. Thus, the participants should not hesitate to report their honest thoughts and feelings. Then, after approving the consent form and answering questions, participants received a compensation of 0.30 $ in exchange of their participation in the survey.

### Measures

The Behavioral Regulation Exercise Questionnaire-2 (BREQ-2, Markland and Tobin, 2004) was used to measure MPE through the 4-item intrinsic regulation (e.g., “Because I think exercise is fun,” α = 0.92), the 3-item identified regulation (e.g., “Because I value the benefits of exercise,” α = 0.81), the 4-item introjected regulation (e.g., “Because I feel guilty when I don’t exercise,” α = 0.75), the 4-item external regulation (e.g., “Because other people say I should,” α = 0.85), and the 4-item amotivation subscales (e.g., “I don’t see why I should have to exercise,” α = 0.88). A-MPE was assessed through different subscales, such as intrinsic and identified regulations, while C-MPE was assessed through the subscales of introjected regulation and external regulation. Impersonal MPE was assessed through the amotivation subscale. The items of BREQ-2 were scored from 1 (“*Not true for me*”) to 5 (“*Very true for me*”).

The 13-item questionnaire provided by Tangney et al. (2004) was used to assess TSC (e.g., “I am good at resisting temptation;” α = 0.76) on a 7-Lickert scale ranging from “*Not at all*” (“1”) to 7 “*Very much so*” (“7”). SWB was assessed through two scales: Happiness and vitality. The 8-item Oxford Happiness Questionnaire (OHQ; Hills and Argyle, 2002) was employed to measure happiness (e.g., “I am well satisfied about everything in my life;” α = 0.81; 1 = “*Strongly disagree*,” 6 = “*Strongly agree*”), while vitality was measured using the 6-item vitality scale of Bostic et al. (2000), which was a revised version of the vitality scale developed by Ryan and Frederick (1997) (e.g., “I feel alive and vital”; α = 0.94; 1 = “*Not at all*,” 7 = “*Very true*”).

### Analysis

To examine our hypotheses, a two-step analysis was carried out in order to assess the quality of model: Measurement model analysis and structural model analysis. Those assessments were carried out using the PLS (Partial Least Square) structural equation method (XLSTAT-PLS, Addinsoft, version 2016.02.29253). A bootstrapping with 1000 iterations of resampling was conducted.

#### Measurement Model and Correlation Analyses

A measurement model includes latent and manifest variables. According to Vinzi et al. (2010), a set of manifest variables can be considered as a latent variable when, at least, one of the following conditions is satisfied: (a) the principal component analysis reveals that the first eigenvalue of the correlation matrix is higher than 1, while the other eigenvalues are smaller; (b) the Cronbach’s alpha index (determining the internal consistency) is larger than 0.700; and (c) the Dillon-Goldstein’s rho index (determining the composite reliability of the latent variables) is larger 0.700 (Vinzi et al., 2010). According to the self-determination theory (e.g., Deci and Ryan, 2008a,b), human functioning can be conceptualized through the concepts of autonomous and controlled motivations, which might both endorse the status of latent variable. As a result, the measurement model considered intrinsic and identified regulations as the manifest variables of the latent variable “A-MPE,” while it considered introjected and external regulations as the manifest variables of the latent variable “C-MPE.” Moreover, the model gathered the constructs of happiness and vitality to form an index of SWB. Furthermore, based on the latent variables coming from confirmatory factor analysis, non-parametric (Spearman’s rho) correlations were carried out.

#### Structural Model and Mediation Analyses

**FIGURE 2:**
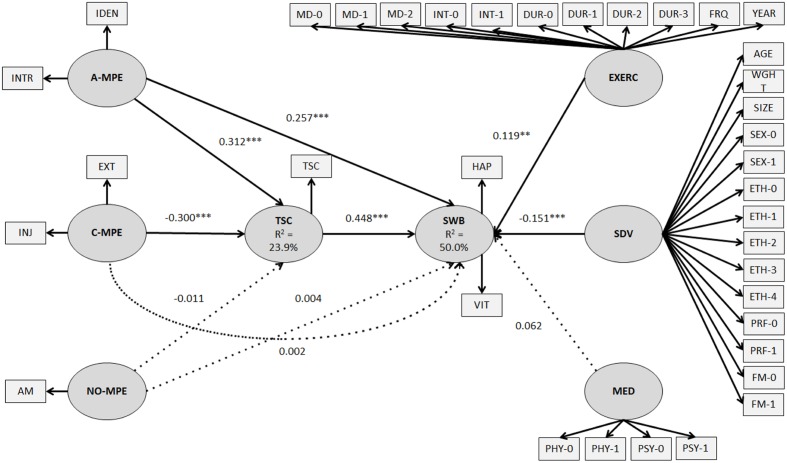
**Structural equation model of the relationships among MPE, TSC (as mediator), and SWB (while controlling for socio-demographic, medical, and exercise variables)**. All coefficients are standardized and solid lines indicate statistical significance. Significance thresholds for a two-tailed test are ^∗∗∗^*p* < 0.001, ^∗∗^*p* < 0.01. A-MPE, autonomous motivation toward physical exercise; INTR, intrinsic regulation; IDEN, identified regulation; C-MPE, controlled motivation toward physical exercise; INJ, introjected regulation; EXT, external regulation; NO-MPE, impersonal motivation toward physical exercise; AM, amotivation; TSC, trait self-control; SWB, subjective wellbeing; HAP, happiness; VIT, vitality; EXERC, exercise-related variables; MD, mode of exercise; INT, intensity of exercise; DUR, duration of exercise; FRQ, frequency of exercise per week; YEAR, number of years of performing exercise regularly; SDV, socio-demographic variables; WGHT, weight; ETH, ethnicity; PRF, professional status; FM, familial status; MED, medical variables; PHY, chronic physical disease; PSY, chronic psychological disease.

A structural model provides standardized path coefficients (estimated through ordinary least squares regressions) that indicated the strength of the causal relationships. Mediation might be shown when the following criteria are satisfied: (a) the direct relationship between independent and dependent variables (excluding the interaction of the mediator) is significant; (b) the mediator establishes significant relationships with the independent and dependent variables; and (c) the indirect and total effects (including the interaction of the mediator) are significant. Additionally, the strength of mediation is assessed through variance accounted for (VAF): VAF = (indirect effect/total effect). The VAF values above 80%, between 20 and 80%, or below 20% indicate that the mediation is full, partial, or non-significant, respectively (Hair et al., 2014). The data were controlled for socio-demography, medical status, and characteristics of exercise as potential confounders (**Figure 2**).

#### Estimation of Model Quality

Two sorts of index were used to assess the quality of measurement and structural models: GoF indexes and the coefficient of determination of the endogenous latent variables, R^2^. Firstly, different GoF indexes can be distinguished: Absolute GoF (assessing the overall quality of the measurement and structural models), relative GoF (corresponding to a transformation of the absolute GoF), outer model GoF (assessing the quality of the measurement model), and inner model GoF (assessing the quality of the structural model). A GoF index varies between 0 (model rejection) and 1 (model validation). More specifically, the critical value for the relative GoF, outer model GoF, and inner model GoF indexes is 0.900. In other words, a value equal to or higher than that threshold for those indexes is considered as satisfying (e.g., Tenenhaus et al., 2005; Vinzi et al., 2010). In addition, a value of the absolute GoF equal to or higher than 0.010, 0.250, or 0.360 reflects a small, moderate, or large overall quality of the both measurement and structural models (Antioco et al., 2008). Secondly, a structural model can be also assessed through *R*^2^ (e.g., Henseler et al., 2009). More specifically, the *R*^2^ values of 0.19, 0.33, and 0.67 are considered weak, moderate, and substantial, respectively (Chin, 1998).

## Results

### Factor Analysis and Correlations

The factor analysis yielded three factors: A-MPE, C-MPE, and SWB (**Table 2**; **Figure 2**). Non-parametric correlations revealed that the three types of motivation (i.e., A-MPE, C-MPE, and NO-MPE) were all related to each other (ρs = -0.513 to 0.511; **Table 3**). A-MPE was negatively related to C-MPE (ρ = -0.233) and NO-MPE (ρ = -0.513), while C-MPE and NO-MPE were positively related (ρ = 0.511; **Table 3**). Furthermore, A-MPE, TSC, and SWB appeared to be all positively related to each other (ρs = 0.382–0.603; **Table 3**). C-MPE and NO-MPE were both negatively related to TSC and SWB (ρs = -0.426 to -0.292; **Table 3**).

**Table 2:** Unidimensionality of manifest variables blocks.

LV Name	# of MVs	Cronbach’s α	D.G.’s ρ	PCA eigenvalues
A-MPE	2	0.700	0.869	1.538
				0.462
C-MPE	2	0.462	0.788	1.300
				0.700
SWB	2	0.866	0.937	1.764
				0.236

*LV, latent variable; MV, manifest variable; D.G.’s ρ, Dillon-Goldstein’s rho; PCA, principal component analysis; oxA-MPE, autonomous motivation toward physical exercise; oxC-MPE, controlled motivation toward physical exercise; SWB, subjective wellbeing*.

**Table 3:** Non-parametric (Spearman’s rho) correlations for all latent variables.

Latent variable	1	2	3	4
1. A-MPE	-			
2. C-MPE	-0.233***	-		
3. NO-MPE	-0.513***	0.511***	-	
4. TSC	0.382***	-0.426***	-0.390***	-
5. SWB	0.516***	-0.292***	-0.358***	0.603***

*The data of all latent variables come from confirmatory factor analysis. ^∗∗∗^*p* < 0.001 for a two-tailed test*.

*A-MPE, autonomous motivation toward physical exercise; C-MPE, controlled motivation toward physical exercise; NO-MPE, no motivation toward physical exercise; TSC, trait self-control; SWB, subjective wellbeing*.

### Structural Equation Model and Mediations

The R^2^ value associated with the endogenous latent variables was moderate, being equal to 36.947% (absolute GoF = 0.330; relative GoF = 0.942; outer model GoF = 0.935; inner model GoF = 1.008; **Table 4**; **Figure 2**). Two significant mediations were found: TSC appeared to mediate the relationships between A-MPE and SWB (partial mediation), and between C-MPE and SWB (full mediation; **Table 5**; **Figure 2**). However, TSC did not mediate the relationship between NO-MPE and SWB (**Table 5**; **Figure 2**).

**Table 4:** Path estimates of the PLS model.

Effects	Path	β	*SE*	*t*-values	*p*-values	*f^2^*
Direct	A-MPE → SWB	0.453	0.058	7.830	0.000	0.196
	C-MPE → SWB	0.151	0.054	2.804	0.005	0.025
	NO-MPE → SWB	0.021	0.063	0.331	0.741	0.000
Mediating	A-MPE → TSC	0.312	0.060	5.244	0.000	0.088
	C-MPE → TSC	0.300	0.055	5.464	0.000	0.095
	NO-MPE → TSC	0.011	0.064	0.169	0.866	0.000
	TSC → SWB	0.448	0.047	9.477	0.000	0.291
	A-MPE → SWB	0.257	0.052	4.961	0.000	0.080
	C-MPE → SWB	-0.002	0.047	-0.033	0.974	0.000
	NO-MPE → SWB	0.004	0.053	0.082	0.935	0.000

*A-MPE, autonomous motivation toward physical exercise; C-MPE, controlled motivation toward physical exercise; NO-MPE, impersonal motivation toward physical exercise; TSC, trait self-control; SWB, subjective wellbeing*.

**Table 5:** Mediation analysis.

Effects	Path	Mediator	IV → Mediator	Mediator → DV	Direct effect	Indirect effect	Total effect	VAF	Mediation strength
Direct without mediator	A-MPE → SWB	N/A	N/A	N/A	0.453^∗∗∗^	N/A	N/A	N/A	N/A
	C-MPE → SWB	N/A	N/A	N/A	0.151^∗∗^	N/A	N/A	N/A	N/A
	NO-MPE → SWB	N/A	N/A	N/A	0.021	N/A	N/A	N/A	N/A
Indirect with mediator	A-MPE → SWB	TSC	0.312^∗∗∗^	0.448^∗∗∗^	0.257^∗∗∗^	0.140^∗∗∗^	0.396^∗∗∗^	35.2%	Partial
	C-MPE → SWB	TSC	-0.300^∗∗∗^		-0.002	0.134^∗∗∗^	0.133^∗^	101.2%	Full
	NO-MPE → SWB	TSC	-0.011		0.004	0.005	0.009	N/A	N/A

*Significance thresholds for a two-tailed test are ^∗∗∗^*p* < 0.001, ^∗∗^*p* < 0.01, ^∗^*p* < 0.05. VAF > 80% = Full mediation, 20% ≤ VAF ≤ 80% = Partial mediation, and VAF < 20% = No mediation (none)*.

*IV, independent variable; DV, dependent variable; VAF, variance accounted for; N/A, not applicable; A-MPE, autonomous motivation toward physical exercise; C-MPE, controlled motivation toward physical exercise; NO-MPE, impersonal motivation toward physical exercise; TSC, trait self-control; SWB, subjective wellbeing*.

## Discussion

The present study utilized the self-determination theory (e.g., Deci and Ryan, 2008a), the control-process theory of self-regulation (e.g., Carver and Scheier, 1998; McCullough and Willoughby, 2009), and the theory of multiple pathways of TSC (e.g., Hagger, 2014) in order to examine how MPE, TSC, and SWB might be interrelated, and whether TSC might mediate the relationships between MPE and SWB.

### Relationships between Motivation toward Physical Exercise, Trait Self-Control, and Subjective Wellbeing

The analyses revealed that A-MPE was positively related to SWB, whereas C-MPE and NO-MPE were both negatively related to SWB. These results support the predictions of the self-determination theory according to which A-MPE (or C-MPE and NO-MPE) may promote (or hinder) wellbeing (Deci and Ryan, 2008a,b). They also support previous studies that showed that autonomous (or controlled and impersonal) forms of MPE were positively associated with positive (or negative) psychological outcomes (e.g., Edmunds et al., 2006; Gillison et al., 2006, 2011; Sebire et al., 2009). According to the self-determination theory, people may deeply experience wellbeing when their social environment supports their innate needs for competence, relatedness, and especially autonomy. By contrast, when the social environment thwarts their need for autonomy, they are more likely to experience a decreased sense of wellbeing or even depression symptoms.

The analyses revealed that A-MPE was positively related to TSC, supporting the results of previous studies that have revealed positive associations between autonomous motivation and indicators of high self-control, such as TSC (Briki et al., 2015), implementation planning (Koestner et al., 2008), and automatic attraction toward helpful goals (Milyavskaya et al., 2015). This result is also compatible with the studies that have shown that autonomous motivation was negatively associated with indicators of low self-control, such as automatic attraction toward temptations and perception of encountering obstacles (Milyavskaya et al., 2015). Finally, our result supports the general view that autonomous activity would improve the effectiveness of self-regulation processes (e.g., goal selection) because such an engagement in the activity would lie in a strong sense of volition and willingness (Carver and Scheier, 1998; Deci and Ryan, 2000). Furthermore, the analyses revealed that C-MPE was negatively related to TSC, running counter most of the studies that have shown independent relationships between controlled motivation and indicators of high self-control (i.e., TSC, implementation planning, automatic attraction toward helpful goals; Koestner et al., 2008; Briki et al., 2015; Milyavskaya et al., 2015). However, our result is consistent with Milyavskaya et al.’s (2015) study that has revealed a positive association between controlled motivation and low self-control (i.e., perception of encountering obstacles). More generally, our result is compatible with the view that controlled activity would lead to a decreased sense of self-regulation because imposed contingencies driving such a psychological functioning would stay away from the self (Carver and Scheier, 1998; Deci and Ryan, 2000). Taken together, our results support the studies that have revealed that autonomous motivation saved more resources and enabled people to perform better on subsequent tasks than controlled motivation (Moller et al., 2006; Muraven et al., 2007, 2008; Muraven, 2008). Furthermore, NO-MPE was negatively associated with TSC, and this result supports the view of the self-determination theory that NO-MPE would be associated with passivity (the opposite of self-control), anxiety, and depression, caused by the dissatisfaction of the three innate needs (e.g., Deci and Ryan, 2008a,b).

The analyses revealed that TSC was positively related to SWB, supporting the results of previous studies that have reported positive associations between self-control, goal attainment, and satisfaction (e.g., Rhodes et al., 2002; De Ridder et al., 2012; Cheung et al., 2014; Hofmann et al., 2014; Briki et al., 2015). More specifically, research has found that TSC: (a) increased positive affect and SWB (e.g., De Ridder et al., 2012; Hofmann et al., 2014; Briki et al., 2015); (b) inhibited conflict among goals and goal-disruptive impulses (e.g., Hagger, 2014; Hofmann et al., 2014); and (c) stimulated and inhibited helpful (e.g., promotion focus) and unhelpful (e.g., prevention focus) plans for action, respectively (e.g., Cheung et al., 2014; Hagger, 2014). Taken together, those studies suggest the view that TSC would develop a strong sense of SWB by optimizing self-regulation processes and increasing the frequency of positive emotions.

### The Mediating Effects of Trait-Self-Control

The analyses revealed two significant mediations. Firstly, TSC appeared to mediate partially the relationship between A-MPE and SWB, supporting the results of Briki et al.’s (2015) study showing a partial mediation of TSC in the relationship between autonomous religious motivation and SWB. More specifically, our result indicates that A-MPE would increase directly SWB, supporting the view that the satisfaction of people’s innate needs would be, by nature, a deep source of wellbeing (e.g., Deci and Ryan, 2008a,b). Our result also indicates that A-MPE would increase indirectly SWB via TSC. This suggests that A-MPE would improve self-regulation processes, thereby leading to facilitate the movement toward the desired goals and to increase the frequency of positive experiences (Koestner et al., 2008; Hofmann et al., 2014; Milyavskaya et al., 2015). Secondly, TSC appeared to mediate fully the relationship between C-MPE and SWB, suggesting that C-MPE would influence SWB only through TSC. In other words, C-MPE would decrease SWB by decreasing the effectiveness of self-regulation processes while pursuing goals (e.g., Deci and Ryan, 2000). Our result runs counter the results of Briki et al.’s (2015) study showing that TSC did not mediate the relationship between controlled religious motivation and SWB, but is compatible with the study of Milyavskaya et al. (2015) showing that self-control mediated the relationship between controlled motivation and goal process. Such inconsistencies should incite psychologists to pay more attention to the link between controlled forms of motivation and the mechanisms of self-control. Furthermore, TSC did not mediate the relationship between NO-MPE and SWB, and NO-MPE did not predict TSC and SWB. These results suggest that NO-MPE would not activate any regulatory processes. Indeed, NO-MPE reflects a psychological state characterized by the absence of intentionality to behave in the exercise context, thus standing in contrast to A-MPE and C-MPE. More generally, this study supports the views that TSC reflects an effective mediating variable to account for the relationship between commitment to an activity and wellbeing (McCullough and Willoughby, 2009), and that TSC corresponds to a crucial determinant of psychological health (e.g., Tangney et al., 2004).

## Conclusion and Perspectives

The results of the present study are the first to support the hypothesis that MPE can influence SWB through TSC. However, this study is not without limitations. Chief among them is its correlational nature, and thus further studies should use stronger causal tests. To do so, experimental studies should examine the effects of stimuli related to A-MPE (e.g., autonomy-supportive context in exercise settings) and C-MPE (e.g., controlling context in exercise settings) on self-control and SWB. Furthermore, by considering motivation, self-control, and feelings as dynamical processes, time-based designs should be used to examine how MPE, TSC, and SWB may fluctuate over time. In that regard, the use of the ambulatory assessment methodology (i.e., computerized devices designed to collect self-reported, physiological, and behavioral data in natural contexts) might be helpful to collect data over time within natural contexts (e.g., Kanning et al., 2012). Importantly, such research directions should shed the light on the mediating role of self-control in the MPE-SWB relationships. From an applied standpoint, in order to promote the development of SWB in exercisers, exercise instructors should promote the development of A-MPE since A-MPE appeared to predict SWB (directly as well as indirectly through TSC). To do so, exercise instructors should enhance exercisers’ perceptions of autonomy by offering them the opportunity of choice, encouraging them to explore new tasks and techniques, listening to them, limiting controlling self-talk, etc. (Standage and Ryan, 2012). In addition, exercise instructors should always associate exercise with the notion of pleasure.

## Author Contributions

WB conceived the study, collected and analysed the data, and wrote and revised the article.

## Conflict of Interest Statement

The author declares that the research was conducted in the absence of any commercial or financial relationships that could be construed as a potential conflict of interest.

## Footnotes

1 www.clickworker.com
